# Adhesive Strips vs. Sutures in Lumbar Spinal Fusion: A Non-inferiority Analysis of Surgical Site Infections

**DOI:** 10.7759/cureus.82065

**Published:** 2025-04-11

**Authors:** Fernando A Núñez-Moreno, Christian Bepperling, Carlos Trenado, Steffen Eitelbuss, Karen Velázquez, Vasilis Karantzoulis, Maria C Ochoa Estrada, Edgar Santos, Farzam Vazifehdan

**Affiliations:** 1 Spine Center, Diakonie-Klinikum Stuttgart, Stuttgart, DEU; 2 Institute of Clinical Neuroscience and Medical Psychology, Medical Faculty, Heinrich Heine University, Düsseldorf, DEU; 3 Faculty of Nursing, Universidad Juárez del Estado de Durango, Durango, MEX

**Keywords:** adhesive strips, adhesive tape, spinal fusion, spondylodesis, surgical site infections, wound infections

## Abstract

Introduction

Surgical site infections (SSIs) continue to pose a significant challenge in spine surgery. Skin closure technique has been shown to impact SSI rates, with sutures traditionally considered the standard. Recently, alternative methods, such as adhesive strips, have gained attention for their potential advantages, including shorter operative times, fewer postoperative visits, and improved cosmetic outcomes. However, their efficacy in managing large surgical wounds, particularly in spine procedures, remains underexplored. This study compares SSI rates between sutures and adhesive strips in posterior one- and two-level lumbar spinal fusion using a non-inferiority design.

Methods

This single-center retrospective study evaluated SSI rates following lumbar spinal fusion using either sutures or adhesive strips for skin closure. Patients who underwent one- or two-level lumbar fusion via an open posterior approach between 2019 and 2022 were initially identified (n = 1,051). Following the application of predefined exclusion criteria, 997 patient records were eligible for review. Propensity score matching (PSM) was then employed to balance key covariates, such as age, sex, ASA classification, surgical time, use of drainage, and the number of levels fused, resulting in 608 patients evenly allocated into two groups. Data were extracted from a prospectively maintained institutional database, and SSIs were classified using the German Krankenhaus Infektions Surveillance System (KISS). A non-inferiority margin of 3% (absolute risk difference) was applied to compare SSI rates. Statistical analyses included t-tests, Mann-Whitney U tests, chi-square tests, and a Wald test for non-inferiority, with the non-inferiority established if the upper bound of the one-sided 95% confidence interval (CI) remained below the 3% margin.

Results

Following PSM, infection rates within a 30-day postoperative follow-up were 1.64% in the suture group and 1.97% in the adhesive strip group. A one-sided Wald test, using a 3% non-inferiority margin, yielded an absolute risk difference of 0.33% (SE = 1.1%; z = -2.47; one-sided p = 0.0067), with the upper bound of the 95% CI at 2.11%.

Conclusion

Adhesive strips demonstrated non-inferiority to sutures in terms of SSI rates following one- or two-level lumbar spinal fusion and may offer the added benefit of reduced operative time. However, further research is warranted to confirm these findings and support the broader implementation of adhesive strips in spinal fusion procedures.

## Introduction

Surgical site infections (SSIs) are among the most common complications in spine surgery, with a meta-analysis estimating an average SSI rate of approximately 2.7% for lumbar spine procedures [1]. In lumbar spinal fusion, reported incidence ranges from 1.9% to 4.48%, influenced by patient characteristics and other risk factors [2-4].

Skin closure technique in posterior spine surgery has been shown to impact SSI rates [5]. Sutures remain the standard against which other methods are compared. Emerging alternatives, such as staples and tissue adhesives, have demonstrated comparable infection rates, along with potential benefits like faster closure and shorter hospital stays [5-7]. Among emerging wound closure methods, adhesive strips (Omnistrip™; Hartmann, Sontheim, Germany) are a form of adhesive tape that has received limited evaluation in large wounds in spinal surgery. At our institution, adhesive strips have been in use since 2017, with wider adoption during the COVID-19 pandemic to reduce patient-healthcare worker interaction. Observed benefits, including shorter surgical duration, fewer postoperative visits, and improved cosmetic outcomes without increased wound-related complications, led to their expanded use in larger wounds. This study aimed to compare SSI rates between sutures and adhesive strips in posterior one- and two-level open lumbar spinal fusion.

## Materials and methods

Study design

This retrospective single-center study employed propensity score matching (PSM) and a non-inferiority analysis to compare sutures and adhesive strips for skin closure, with SSI rates as the primary outcome. A single brand of adhesive strips (Omnistrip™) was used throughout the study.

The study population included patients who underwent one- or two-level lumbar spinal fusion via an open posterior approach at the Spine Center of Diakonie-Klinikum Stuttgart. Data were collected from 2019 to 2022 using the institution’s prospectively maintained database, which systematically tracks SSIs in treated patients. This study was approved by the Ethics Commission of the State Medical Association of Baden-Württemberg (F-2024-077).

Postoperative follow-up was conducted through structured consultations by the attending surgeon to evaluate the patient’s clinical status and wound healing. During these consultations, the surgical wound was carefully assessed for signs of infection. If any signs were detected, the patient underwent a wound culture to confirm the presence of an infection. SSIs were diagnosed based on clinical signs and subsequently confirmed by positive wound cultures. The severity of infection was classified according to the German Krankenhaus Infektions Surveillance System (KISS), which categorizes infections based on the depth of tissue involvement. Superficial infections (A1) are confined to the skin and subcutaneous tissues, deep infections (A2) extend into the deeper soft tissues such as fascia and muscle, and organ/space infections (A3) involve any part of the anatomy (e.g., an organ or body cavity) that was manipulated during the surgical procedure [8].

Study population

Patients included in the analysis underwent open posterior spinal fusion surgery involving one or two vertebral segments, with skin closure performed using either absorbable or nonabsorbable sutures, or adhesive strips. During the follow-up period, 1,051 patients were identified. Exclusion criteria included patients younger than 18 years; those with pre-existing SSIs (e.g., spondylodiscitis); surgical procedures other than open posterior spinal fusion; revision surgeries due to non-wound-related complications (e.g., pedicle screw or interbody cage fractures); endoscopic approaches; and surgeries involving more than two vertebral segments. Applying these criteria resulted in the exclusion of 36 records. Additionally, 13 patients with infections classified as A3 were excluded, resulting in a total of 49 records being eliminated based on the exclusion criteria. Finally, six more patients were removed due to missing data, yielding an overall exclusion of 55 records.

Only SSIs occurring within 30 days postoperatively were analyzed. The analysis specifically focused on infections categorized as A1 and A2, as infections classified as A3 (organ/space) were unlikely to be significantly influenced by the skin closure technique.

Surgical technique

**Figure 1:**
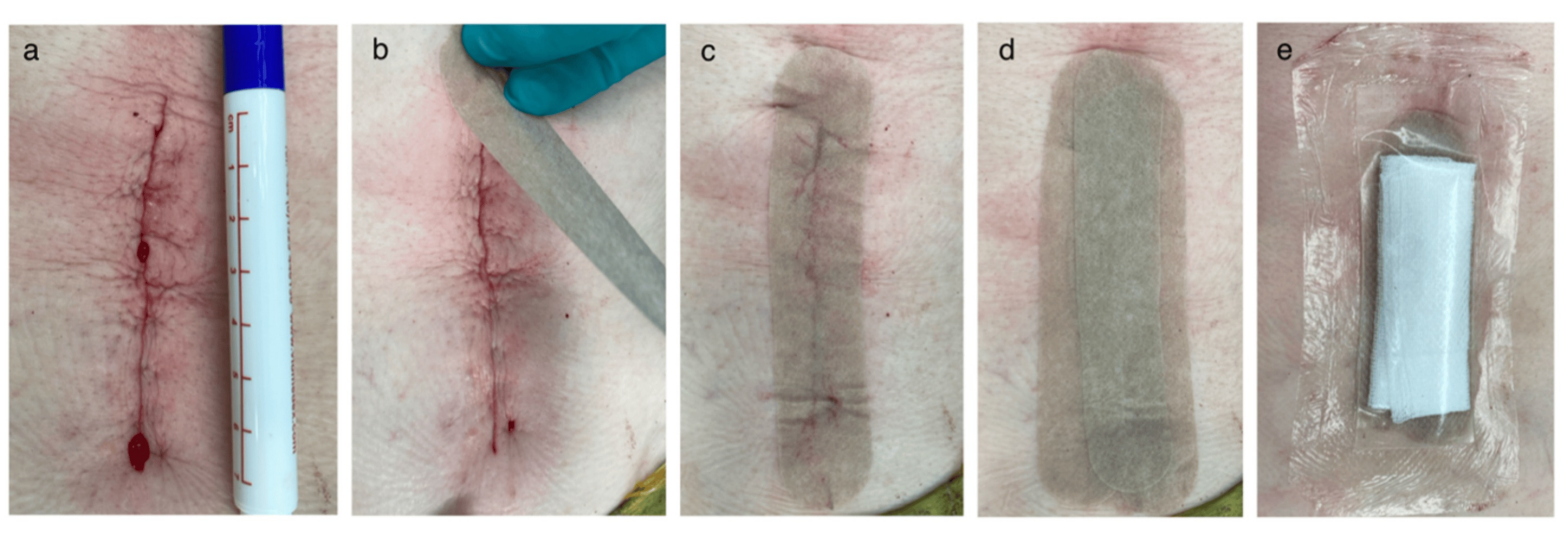
Adhesive strip application technique. (a) Subdermal approximation of the wound with interrupted suture of a wound of approximately 7 cm long. (b) Placement of adhesive strips in the surgical wound. (c) Surgical wound totally covered with adhesive strips. (d) Second layer of adhesive strips applied to the covered wound. (e) Wound covered with gauze dressing for absorption of excess blood and covered with a transparent dressing.

The surgical technique included fascial closure and subcutaneous tissue approximation, both performed using a standard interrupted suture technique with absorbable sutures in both groups. For skin closure, two methods were used: in the suture group, wounds were closed using either interrupted transcutaneous stitches with nonabsorbable sutures or an absorbable subcuticular technique; in the adhesive strip group, wound edges were manually approximated before the sequential application of adhesive strips (Omnistrips) (Figure 1).

All procedures were conducted by a fixed and experienced surgical team. The department performs over 2,000 spine surgeries annually, with each surgeon operating 3-4 days per week, and a senior surgeon always present during the procedures. Although residents assist during surgeries, they are never solely responsible for skin closure.

Following final skin closure, whether achieved with sutures or adhesive strips, the wound was covered with sterile gauze and a transparent dressing, with dressing changes performed only in cases of postoperative bleeding. All patients received a single preoperative dose of intravenous cefazolin as antibiotic prophylaxis, and no postoperative antibiotics were administered. A closed, rigid Redon drain was placed at the discretion of the operating surgeon and was removed when drainage was less than 100 mL/24 hours, typically after 48 hours. Notably, the use of adhesive strips increased progressively throughout the study period.

Propensity score matching

The data were analyzed prior to matching to obtain general insights. To minimize selection bias in the final analysis, a PSM approach was applied. The propensity score was estimated using a logistic regression model, with the type of wound closure material as the dependent variable.

Covariates included gender, age, ASA classification, surgical time, use of drainage, and the number of surgical segments involved. Nearest neighbor matching without replacement was performed using a caliper width equal to 0.2 times the standard deviation of the logit of the propensity score. After matching, all standardized mean differences (SMDs) were below 0.15, indicating an acceptable balance between the groups: gender (SMD = 0.1092), age (SMD = 0.0007), surgical time (SMD = -0.1466), use of surgical drains (SMD = 0.1244), and number of segments (SMD = -0.0667).

Statistical analysis

All statistical analyses and graphical representations were performed using IIBM SPSS Statistics for Windows, Version 25.0 (Released 2017; IBM Corp., Armonk, NY, United States) (RRID:SCR_002865), R (R Foundation for Statistical Computing, Vienna, Austria, https://www.R-project.org/) (RRID:SCR_001905), and Matlab Version 9.12 (RRID:SCR_001622). Frequencies and proportions were calculated along with their 95% confidence intervals (CIs). To assess the normality of quantitative data, a Shapiro-Wilk test was performed. Based on the results, an independent t-test was employed for normally distributed data, while the Mann-Whitney U test was used for nonparametric data. Categorical data were analyzed using the chi-square test, and ordinal data were evaluated with the Cochran-Armitage test for trend. The null hypothesis was rejected if the p-value was less than 0.05.

Non-inferiority margin

Non-inferiority was assessed using a Wald test, with a 3% non-inferiority margin for absolute risk differences. This margin was chosen based on a review of relevant non-inferiority trials and clinical considerations [9,10]. Although some studies in spine surgery have used margins as high as 10% [11,12], our literature review indicated an anticipated infection rate between 2% and 5%, leading us to select a more conservative margin. This approach was designed to balance statistical power with clinical relevance, thereby minimizing the risk of erroneously declaring non-inferiority when a clinically meaningful difference might exist. The null hypothesis was rejected if the upper bound of the one-sided 95% CI for the absolute risk difference remained below the predefined threshold (Ho: p1-p2 ≥ δ p1, adhesive strip group SSI rates; Ha: p1-p2 ≤ δ p2, suture group SSI rates; δ = 0.03).

## Results

A total of 997 patients were included in the analysis, of whom 346 (34%) were treated with sutures and 651 (65%) with adhesive strips.

The baseline characteristics of the population are described in Table 1. In summary, there were statistically significant differences in gender distribution (p = 0.029), surgical time (p < 0.001), hospital stay (p < 0.001), ASA score (p < 0.001), and utilization of surgical drains (p < 0.001).

**Table 1:** Demographic and surgical characteristics before propensity score matching. ASA: American Society of Anesthesiologists Physical Status Classification System, SD: standard deviation. ^a^Surgical time is expressed in minutes. ^b^Length of stay is expressed in days. *Statistically significant difference.

Patient characteristics	Suture	Adhesive strips	p-value
Age (SD)	66.9 (13.11)	66.9 (13.26)	0.27
Gender (%)			0.029*
Female	244 (70.5)	413 (63.4)	
Male	102 (29.5)	238 (36.5)	
Surgical time^a^ (SD)	132.2 (43.25)	116.2 (40.5)	2.34E-08*
Length of stay^b^ (SD)	8.8 (5.03)	7.5 (5.2)	2.31E-20*
Segments (%)			0.065
1	233 (67.3)	476 (73.1)	
2	113 (32.65)	175 (26.8)	
Use of surgical drains (%)			1.01E-16*
Yes	175 (50.5)	499 (76.6)	
No	171 (49.4)	152 (23.3)	
ASA (%)			2.01E-05*
I	15 (4.3)	13 (1.9)	
II	237 (68.4)	380 (58.37)	
III	94 (27.1)	257 (39.4)	
IV	0	1 (0.1)	
V	0	0	
VI	0	0	

To control for differences in the cohort, a PSM analysis was performed. The matching process excluded 347 patients from the adhesive strip group and 42 patients from the suture group. The final cohorts each consisted of 304 patients, and differences between the groups were balanced, with the exception of length of stay (p < 0.001). The characteristics of the matched cohort are shown in Table 2.

**Table 2:** Demographics and surgical characteristics after propensity score matching. ASA: American Society of Anesthesiologists Physical Status Classification System, SD: standard deviation. ^a^Surgical time is expressed in minutes. ^b^Length of stay is expressed in days. *Statistically significant difference.

Patient characteristics	Sutures	Adhesive strips	p-value
Age (SD)	66.9 (13.3)	66.9 (13.1)	0.906
Gender (%)			0.198
Female	209 (68.7)	193 (63.4)	
Male	95 (31.2)	111 (36.5)	
Surgical time^a^ (SD)	127.1 (41.1)	121.2 (43.1)	0.065
Length of stay^b^ (SD)	8.8 (5.1)	7.2 (4.4)	7.92673E-16*
Segments (%)			0.467
1	216 (71.9)	225 (74.0)	
2	88 (28.9)	79 (25.9)	
Use of surgical drains (%)			0.213
Yes	175 (57.5)	191 (62.8)	
No	129 (42.4)	113 (37.1)	
ASA (%)			0.579
I	11 (3.6)	7 (2.3)	
II	199 (65.4)	200 (65.7)	
III	94 (30.9)	97 (31.9)	
IV	0	0	
V	0	0	
VI	0	0	

Surgical site infection (SSI) rates and non-inferiority

**Figure 2:**
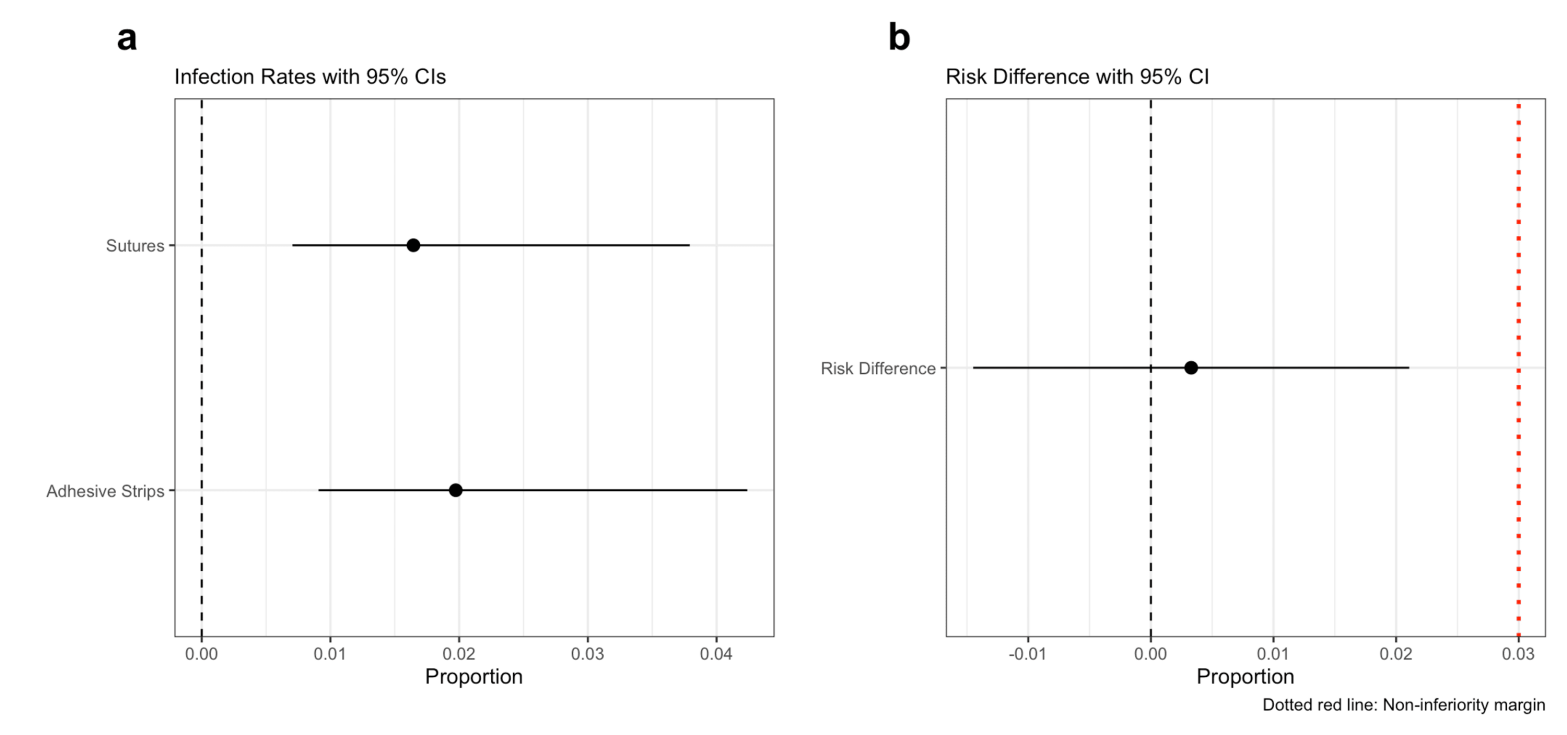
Forest plot of infection rates and absolute risk difference with non-inferiority margin. (a) Infection rates for adhesive strip and suture groups, with 95% CI. (b) Absolute risk difference with its 95% CI. The dotted red line indicates the non-inferiority margin (δ = 0.03). The upper bound of the 95% CI for the absolute risk difference is below the margin of non-inferiority.

After PSM, SSI rates were 1.64% (95% CI 0.70%-3.79%) for the suture group and 1.97% (95% CI 0.91%-4.24%) for the adhesive strip group (Figure 2). The one-sided Wald test using a 3% non-inferiority margin yielded an absolute risk difference of 0.33% (SE = 1.1%), with z = -2.47 and a one-sided p-value of 0.0067. The upper bound of the one-sided 95% CI for the absolute risk difference was 2.11%, which is below the predefined 3% margin.

## Discussion

Our results suggest that adhesive strips may represent a viable alternative to sutures for wound closure in open posterior one- and two-level lumbar spinal fusion, with comparable SSI rates. Statistical analysis showed a 0.33% difference in SSI rates, supporting non-inferiority within the predefined 3% absolute risk difference margin.

These findings align with other studies in spinal surgery. For example, Yang et al. conducted a retrospective study of 36 patients undergoing percutaneous vertebroplasty with 1.5-2 cm incisions, in which adhesive tapes were used in 91.6% of cases and sutures in 8.3% [13]. No SSIs were observed in either group, and only one complication due to bleeding occurred in the adhesive tape group [13]. A larger retrospective study sponsored by Ethicon compared the Dermabond Prineo-Skin Closure System (Cincinnati, OH, USA), which integrates 2‑octyl cyanoacrylate with a self-adhesive polymer mesh tape (2OPMT), to skin staples in spinal fusion surgery. Involving 7,204 patients from a multicenter database, the 2OPMT group exhibited reductions in surgical time (240 vs. 270 minutes), length of stay (3.35 vs. 3.86 days), and SSI rates (1.11% vs. 2.07%) [7]. Similarly, a retrospective matched cohort study of 240 patients undergoing multilevel spinal fusion found that using the 2OPMT system in combination with barbed sutures resulted in decreased wound closure time, with comparable wound-related complications at three-month follow-up [14].

While the data on surgical tape in spine surgery consistently suggest comparable SSI rates to traditional closure methods, it is important to note that Yang et al. evaluated only small wounds, and the 2OPMT studies combined tape with a tissue adhesive-limiting direct comparability with our results [13].

Evidence from other surgical fields further supports the use of adhesive strips across various contexts. In coronary artery bypass surgery, Lazar et al. demonstrated that adhesive strips for saphenous vein harvesting were associated with reduced early postoperative pain, shorter closure times, and comparable wound-related complications compared to subcuticular sutures and skin sealants [15]. In a separate trial, adhesive strips were shown to be the fastest method for closing a median sternotomy without increasing wound-related complications [16]. Additional randomized trials in episiotomy closure, thyroid/parathyroid surgery, and pediatric groin wound closure have similarly reported that adhesive strips result in reduced postoperative pain, equivalent cosmetic outcomes, and similar complication rates compared to sutures [17-19].

Meta-analyses further underscore the advantages of adhesive strips. Luo et al. found that, in total knee arthroplasty, adhesive strips yielded comparable wound complication rates while reducing readmission rates relative to surgical staples [20]. Likewise, a meta-analysis of 18 randomized trials in thyroid and parathyroid surgeries reported that adhesive strips provided the fastest wound closure, with complication rates similar to those observed with metal clips and sutures [21].

Despite generally favorable outcomes, some studies have reported a higher incidence of wound-related complications, such as bleeding and dehiscence, with the use of adhesive strips. For example, Yang et al. observed that one patient (3%) in the adhesive strip group experienced significant bleeding that required wound evacuation and reapplication of the tape [13]. Similar concerns have been documented in abdominal surgery, where adhesive strips were associated with increased rates of dehiscence and bleeding compared to sutures [22,23]. These discrepancies may be attributed to variations in closure techniques, including the omission of subcutaneous suturing in some minimally invasive procedures.

In addition, adhesive strips may provide benefits by minimizing direct wound contact and reducing percutaneous bacterial entry, potentially resulting in a lower inflammatory response, as suggested by animal studies [24]. They can also reduce surgical time, a factor known to correlate with decreased SSI risk; a meta-analysis across various surgical disciplines demonstrated a 13% increase in SSI likelihood for every additional 15 minutes of operative time [25]. In our pre-PSM data, we observed a reduction of nearly 15 minutes in operative time, a difference that may be particularly clinically significant in more extensive procedures. Although the upfront cost of adhesive strips is higher, these expenses may be offset by the reduction in surgical time and decreased staff time required compared to traditional suturing methods. In fact, previous research has identified adhesive strips as the most cost-effective alternative to sutures in low-tension wounds [26].

In summary, our findings, as supported by existing literature, indicate that adhesive strips are a viable option for wound closure in spinal fusion surgery, offering comparable SSI rates along with potential advantages such as reduced surgical time and shorter hospital stays. However, other important outcomes, such as surgical wound dehiscence and postoperative bleeding, could not be assessed in this study and should be the focus of future research. Further studies, particularly prospective or randomized trials, are needed to evaluate these potential complications in the context of spine surgery and to strengthen the evidence base supporting the broader use of adhesive strips.

The limitations of this study, inherent to its retrospective design, include incomplete data on key patient risk factors, such as comorbidities and body mass index, which are known to influence SSI rates but were not available in our dataset. Additionally, the study did not assess wound-related complications such as bleeding and dehiscence, which are important clinical outcomes, nor did it evaluate patient satisfaction, a parameter that could have further supported our conclusions. Furthermore, the application of PSM resulted in a smaller sample size, and a temporal trend toward an increased proportion of single-level fusions in later years may have introduced selection bias. These limitations underscore the need for future prospective or randomized studies that incorporate additional key factors to validate and extend our findings.

Nonetheless, our study contributes to the growing body of evidence supporting alternative skin closure methods in spinal surgery and, to our knowledge, is the first to specifically evaluate the use of adhesive strips in large lumbar wounds following spinal fusion. These findings may also have broader applicability to procedures involving smaller incisions, such as lumbar decompression. However, further research is warranted to address the limitations of this study. In particular, prospective studies and randomized controlled trials are needed to provide higher-level evidence supporting the use of adhesive strips in spinal fusion, as such data are currently lacking.

## Conclusions

Our results support the use of adhesive strips as an alternative to sutures in one- and two-level lumbar spinal fusion procedures, demonstrating non-inferior SSI rates. These findings suggest that adhesive strips may be a viable option for skin closure in other spinal surgeries involving similar or smaller incision sizes, potentially offering added benefits such as reduced operative time, comparable cosmetic outcomes, decreased postoperative pain, and greater patient satisfaction, as reported in international literature. However, further research is necessary to confirm the efficacy and safety of adhesive strips in spinal fusion surgery, particularly through prospective or randomized clinical trials that address the limitations of the present study and evaluate complications beyond SSIs.
